# Supplementary material for: Causal interplay between lactose intolerance and gut microbiota: a combined bidirectional Mendelian randomization and in vivo validation study
Source: Front Nutr. 2026 Jun 1;13:1803337. doi: 10.3389/fnut.2026.1803337 (PMC13265576; doi:10.3389/fnut.2026.1803337)
Supplement: Supplementary file 1 [file Data_Sheet_1.zip › supplementary materials/Forward/leave one out cross validation/ebi-a-GCST90027605.finngen_R12_E4_LACTONAS.pdf]

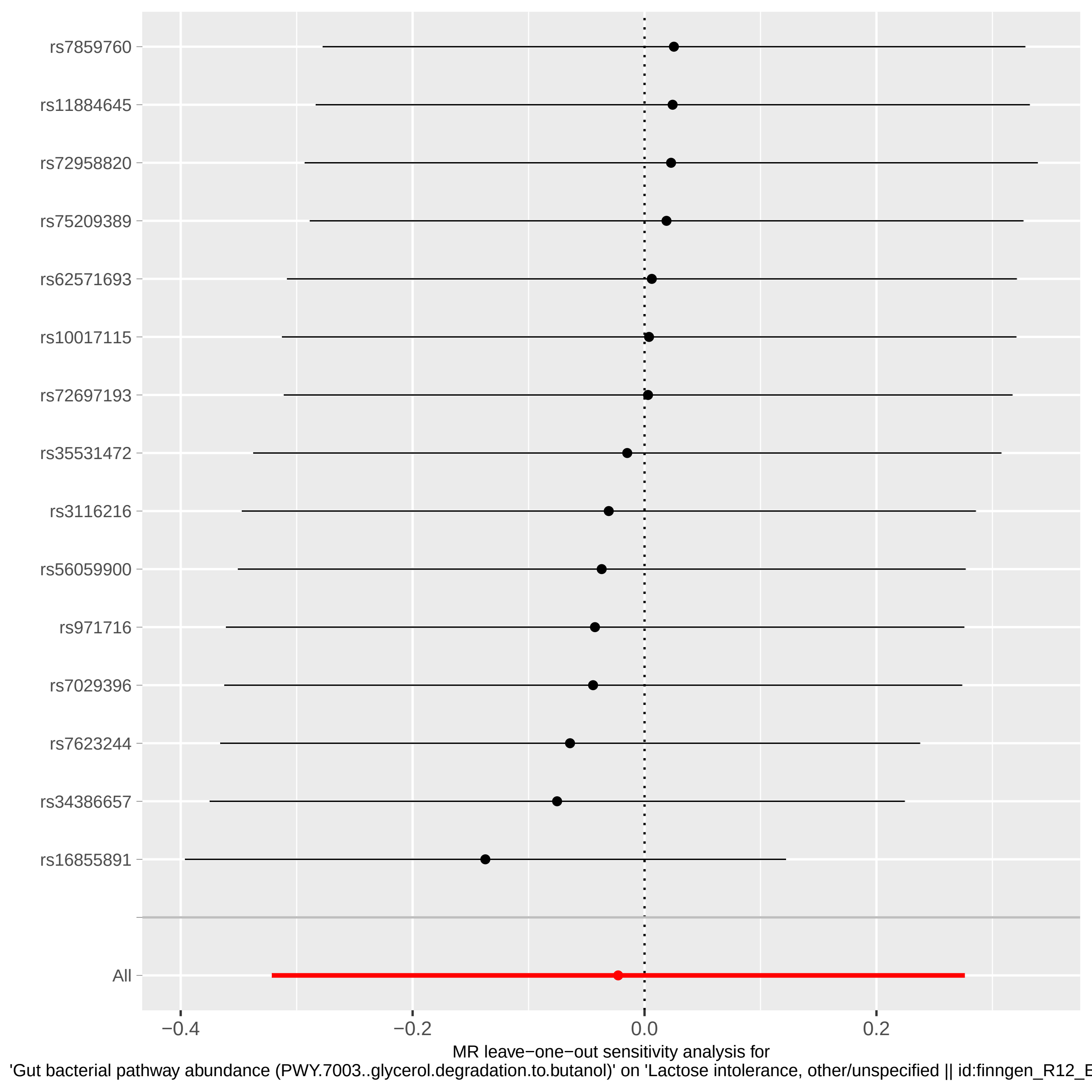

rs7859760
rs11884645
rs72958820
rs75209389
rs62571693
rs10017115
rs72697193
rs35531472
rs3116216
rs56059900
rs971716
rs7029396
rs7623244
rs34386657
rs16855891
All
−0.4
−0.2
0.0
0.2
MR leave−one−out sensitivity analysis for
'Gut bacterial pathway abundance (PWY.7003..glycerol.degradation.to.butanol)' on 'Lactose intolerance, other/unspecified || id:finngen_R12_E
